# Overview of meningococcal epidemiology and national immunization programs in children and adolescents in 8 Western European countries

**DOI:** 10.3389/fped.2022.1000657

**Published:** 2022-11-23

**Authors:** Gaëlle Pinto Cardoso, Marion Lagrée-Chastan, Marion Caseris, Joël Gaudelus, Hervé Haas, Jean-Philippe Leroy, Pierre Bakhache, Jean-François Pujol, Andreas Werner, Marie-Aliette Dommergues, Emilie Pauquet, Didier Pinquier

**Affiliations:** ^1^ Service de Pédiatrie Néonatale et Réanimation, University of Rouen Normandy, CHU Rouen, Hôpital Charles Nicolle, Rouen, France; ^2^ Urgences Pédiatriques et Maladies Infectieuses, CHU Lille, Hôpital Jeanne de Flandre, Lille Cedex, France; ^3^ Hôpital Robert-Debré, AP-HP, Paris, France; ^4^ Service de Pédiatrie, Hôpital Jean Verdier, Bondy Cedex, France; ^5^ Service de Pédiatrie - Néonatalogie, Centre Hospitalier Princesse Grace, Monaco Cedex, Monaco; ^6^ Service des Maladies Infectieuses et Tropicales, DIIM/SIBM, CHU Rouen, Hôpital Charles Nicolle, Rouen, France; ^7^ Cabinet de Pédiatrie, AFPA, Saint-Quentin, France; ^8^ Service de Pédiatrie, Polyclinique Bordeaux-Rive-Droite, Lormont, France; ^9^ AFPA, Villeneuve les Avignon, France; ^10^ Service de Pédiatrie, Centre Hospitalier de Versailles, Le Chesnay, France; ^11^ Unité de Néonatologie, Soins Intensifs Néonataux, Hôpital des Enfants, CHU de Bordeaux, Bordeaux Cedex, France; ^12^ Service de Pédiatrie Néonatale et Réanimation, University of Rouen Normandy, CHU Rouen, Rouen, France

**Keywords:** Europe, invasive meningococcal disease (IMD), meningococcal vaccination, national immunization program (NIP), menACWY vaccination, menB vaccination

## Abstract

**Background:**

In Europe, meningococcal (Men) vaccines are available against 5 of the 6 serogroups responsible of nearly all cases of invasive meningococcal disease (IMD). Meningococcal vaccination has been introduced in the national immunization programs (NIPs) for children and adolescents of numerous European countries, but with no consistent strategy across countries.

**Objectives:**

To describe IMD epidemiology, NIPs, and vaccination coverage rates (VCRs) in children and adolescents in 8 Western European countries.

**Methods:**

Epidemiological data (from 1999 to 2019), NIPs regarding meningococcal vaccination status, and VCRs were collected from the European Centre for Disease Prevention and Control (ECDC) and/or national websites.

**Results:**

MenB was the most common serogroup. In Belgium, Spain, France, the Netherlands, the United Kingdom (UK), and Portugal, incidence was greater for MenW than MenC. In 2019, MenB risk was covered in 2 countries (Italy, UK). MenC risk was covered in all countries, *via* MenC only (countries: *N *= 3), MenACWY only (*N *= 2), or MenC (infants/children) and MenACWY (adolescents) (*N *= 3) vaccination. VCRs were higher in children than adolescents.

**Conclusion:**

Our study confirmed the diversity of NIPs, including in neighboring European countries with similar factors like economic resources and epidemiological risk, thus indicating that other factors underlie NIPs. Convergence toward a more common immunization program including MenACWY and MenB vaccination would promote equity and safe travel regarding infectious diseases for young people, and possibly improve the understanding of vaccination by patients and healthcare professionals.

## Introduction

With 3,233 confirmed cases in European Union/European Economic Area (EU/EEA) member states (30 countries) and 324 deaths in 2018, invasive meningococcal disease (IMD) is an uncommon but life-threatening disease (1). The case fatality rate is usually almost 10% (2). Up to 20% of IMD survivors suffer from disabling sequelae (such as hearing loss, neurologic and cognitive damage, or limb amputation) (2) that may have devastating effects on individuals and their family (3) and significant economic impact on society (4).

IMD affects all age groups, but notification rates (NRs) in developed countries are usually highest in infants (<1 year), followed by toddlers (1–4 years), with a second peak in young people (15–24 years) (5). In 2018, in the EU/EEA member states, the IMD NR was 0.6 cases per 100,000 population: 8.3 for infants, 2.4 for toddlers, and 0.9 for young people (1).

IMD is due to *Neisseria meningitidis*, an aerobic Gram-negative diplococcal commensal bacterium of the human rhino-oropharynx, which in <1%–5% of cases invades the mucosa and enters the bloodstream, causing meningitis and/or sepsis (6). Among the dozen N. meningitidis serogroups identified by the composition of the bacterial capsular polysaccharide, 6 (A, B, C, W, X, and Y) are responsible for virtually all cases of IMD worldwide (7). The relative importance of each serogroup depends on geographic location. In 2019, serogroups B, C, and Y were predominant in North America, serogroups B, C, and W in South America, serogroups B, C, W, and X in Africa, and serogroups A, B, and W in Asia (8). In Europe, serogroups B, C, W, and Y are responsible for more than 95% of IMD cases (7). Since the 1960s, serogroup B has usually predominated; serogroup C emerged in the late 1990s and was the second most prevalent serogroup until 2016; serogroups W and Y are less frequently reported, despite outbreaks caused by serogroup W (9). IMD epidemiology is dynamic (2). In Europe, confirmed cases of IMD with a known serogroup were mainly due to serogroup B (51% of IMD cases overall and 71% of cases in children <5 years in 2018), followed by serogroup W (18% of cases in 2018) and serogroup C (15% of cases in 2018) (1). However, meningococcal serogroup B (MenB) incidence has been declining since 2014, particularly in infants: NR declined from 7.8 to 6.0 cases per 100,000 from 2014 to 2018. During the same period, the MenC NR was stable, ranging between 0.08 and 0.1/100,000, and the MenW NR increased from 0.04 to 0.12/100,000, the highest increase being in children <5 years. For MenY, NR tended to increase, from 0.05 to 0.07/100,000 during the last 5 years (1).

IMD is highly unpredictable (2, 10), although most cases occur in the winter months in Europe (1). It manifests as isolated cases or outbreaks (2). Outbreaks or clusters occur in certain settings, such as universities, due to lifestyle and living conditions (10). Outbreaks also occur when hyperinvasive strains are transmitted within a susceptible population (11).

Finally, the natural tendency of IMD epidemiology, in terms of both incidence and serogroup distribution, to change over time, the phenomenon of secular trends, and the onset of hyperinvasive strains contribute to the abruptness and unpredictability of the disease.

There is no universal vaccine preventing all serogroups causing meningococcal disease but, to date, several vaccines have been developed for the prevention of the main IMD-causing serogroups (12). Over time, 3 types of vaccine have been developed (13, 14). Polysaccharide vaccines were developed in the 1940s; they were safe and effective in children and adults, but weakly protective in infants and toddlers <2 years. Polysaccharide-protein conjugate vaccines (conjugate vaccines) were developed in the 1990s to overcome this limitation. Conjugate vaccines are safe and effective in infants, toddlers, children, and adults. They prevent carriage, reducing transmission and leading to herd protection. They are used as an NIP component *via* monovalent (MenC) or multivalent (MenACWY) formulations. Protein-based vaccines have been developed for MenB, because the MenB capsular polysaccharide was too similar to human neural adhesion molecules to be used as a vaccine antigen. A MenB vaccine made from 4 common proteins found on the surface of MenB meningococcal bacteria, combined with the outer membrane vesicles (OMV) from 1 MenB strain, was approved in Europe in 2013 (15, 16). It protects individuals of all ages against most strains of MenB but has no discernible effect on carriage or transmission (17). According to the World Health Organization (WHO), in July 2018, in addition to the MenA and MenC monovalent conjugate vaccines, 3 MenACWY polysaccharide conjugated vaccines and 3 protein/OMV-based MenB vaccines were licensed (13).

Globally, vaccination policies vary significantly from country to country (13): 43, 14, and 28 NIPs target infants and children, adolescents, and special groups (e.g., the military), respectively. In Europe, MenC vaccination was first introduced in the routine childhood NIP in the UK, in 1999, with a conjugate vaccine. Since then, recombinant protein meningococcal B (MenB) vaccines and quadrivalent (MenACWY) meningococcal conjugate vaccines have been authorized (18). All EU countries approve the WHO vaccination strategy (19), which recommends large scale vaccination in countries experiencing high (>10/100,000 per year) or intermediate (2–10/100,000) endemic rates of IMD, and in countries with frequent epidemics (13). However, at the time of writing, the incidence of IMD was drastically lower (<2/100,000 per year for all serogroups), leading to differences in routine meningococcal vaccination between EU countries, as the benefit/risk-cost balance is less clear and the prevention strategy is difficult to define (20). As of July 2, 2022, 12 of the 30 ECDC countries (21) did not include any meningococcal vaccination in the program for the general population, 8 included MenB and 17 MenC (7 exclusively with MenC vaccines, 4 exclusively with MenACWY vaccines, and 6 with a mix of MenC vaccines for infants and MenACWY vaccines for adolescents). France is the only country that includes MenC prevention for infants and toddlers in a mandatory immunization program. Finally, as indicated by Martinon-Torres et al. (12), meningococcal vaccination has been introduced in the NIPs of several European countries, but with no consistent strategy across countries. Each country uses different vaccines and schedules in different age groups; vaccination recommendations vary with local and regional disease progression and with national healthcare priorities.

By describing the epidemiology of IMD, the different NIPs and VCRs in children and adolescents in 8 Western European countries, our objective was to open the way to a discussion of discrepancies between EU NIPs.

## Material and methods

### Study design and hypothesis

To systematically assess differences in NIPs for children and adolescents in EU countries and the main factors underlying these differences, 11 pediatricians and one infectious disease specialist with strong interest in IMD prevention (the authors of the present article) reviewed NIPs for the 4 main disease-causing serogroups (MenB, MenC, MenW, and MenY) in France and surrounding EU countries, in relation to epidemiology. Vaccination coverage was analyzed, as achieving a high VCR is the key to effective vaccination programs.

Each vaccination strategy was assessed in terms of direct protection against the most frequent serogroups in the appropriate age groups (children and adolescents), indirect protection objectives (herd immunity) *via* carriage prevention in adolescents, and/or presumed upcoming changes in IMD epidemiology.

### Selected European countries

The following countries were selected: Belgium (BE), Germany (DE), Spain (ES), France (FR), Italy (IT), Portugal (PT), the Netherlands (NL), and the UK (UK).

Selection criteria comprised: (i) EU member in 2019, which facilitated data collection; (ii) geographically situated around France, which facilitates interactions that could lead to similar epidemiologies, and (iii) similar per capita gross domestic product (GDP), to avoid bias linked to economic factors influencing recommendations. Luxembourg, which is an EU member neighboring France, was excluded due to its exceptionally high per capita GDP (€85,030 in 2019) (22).

### Data sources

The Eurostat database was used to collect economic and demographic data for selected countries (22).

The latest epidemiological data were obtained from the ECDC website; the ECDC monitors infectious diseases for the EU member states, including for the UK until 2018. NIPs for meningococcal vaccination were obtained from the ECDC and the national UK websites (21, 23). VCRs were obtained from the health authority's website for each country (23–30). Further details are presented in Supplementary Table S1.

### Collected data, data selection and analysis

Epidemiological data from 1999 (date of the introduction of the conjugate MenC vaccine in the UK) to 2019 (before the COVID-19 pandemic) or the latest available data for national meningococcal vaccination recommendations and VCRs were collected for each of the 8 countries.

## Results

### Selected countries

In 2019 (i.e., before Brexit), all countries belonged to the EU-28. Altogether, they included about two-thirds of the EU population, being some of the most highly populated countries. Their per capita GDP was close to the EU-28 average, ranging from €18,670 (PT) to €41,980 (NL). Based on demographic indicators and as compared with other selected countries, Spain and Italy had the oldest population and lowest crude birth and infant mortality rates (Table 1).

**Table 1:** Economics and demographic characteristics of the 8 selected countries in 2019 (22).

Country	Schengen border-free area	Number of inhabitants	Real per capita GDP (€)	Proportion of 0–14-year-olds (%)	Proportion of 15–24-year-olds (%)	Age (years) of mother at birth of first child	Crude birth rate	Infant mortality rate
BE	Yes	11,445,519	36,080	16.9	11.4	29.1	10.2	3.7
DE	Yes	83,019,213	35,950^(*p*)^	13.6	10.4	29.8	9.4	3.2
ES	Yes	46,937,060	25,200^(*p*)^	14.8	9.8	31.1	7.6	2.6
FR	Yes	67,290,471	33,250^(*p*)^	18.0^(*p*)^	11.8^(*p*)^	28.8^(*p*)^	11.2^(*p*)^	3.8
IT	Yes	59,816,673	27,230	13.2	9.8	31.3	7.0	2.4
NL	Yes	17,282,163	41,980	15.9	12.3	30.1	9.8	3.6
PT	Yes	10,276,617	18,670	13.7	10.6	29.9	8.4	2.8
UK	No	66,647,112	32,910	17.9	11.8	29.0^(a)^	10.7	3.9^(b)^
**EU-28**	**–**	**513,206,391**	**28,680**	**15.5^(e)(*p*)^**	**10.8^(e)(*p*)^**	**29.4^(b)(e)(*p*)^**	**9.5^(e)(*p*)^**	3.4^(a)(b)^*^†^
Range	–	(493,559–83,019,213)^1^	(6,630–85,030)^2^	(13.2–20.5)^3^	(8.9–12.8)^4^	(26.3–31.3)^5^	(7.0–12.0)^6^	(1.6–6.7)^7^

BE, Belgium; DE, Germany; ES, Spain; EU, European Union; FR, France; GDP, Gross domestic product; IT, Italy; NL, Netherlands; UK, United Kingdom.

Number of inhabitants: on January 1, 2019; Real GDP per capita: ratio of the real GDP to the average population in 2019 (GDP measures the value of the total final output of goods and services produced by an economy within a certain period); Proportion by age group: share of population in a certain age group compared to the total population; Crude birth rate: ratio of the number of live births during the year to the average population in that year (expressed per 1,000 people); Infant mortality rate: ratio of the number of deaths of children under 1 year of age during the year to the number of live births in that year (expressed per 1,000 live births).

^(a)^2018; ^(b)^27 countries; ^(e)^estimated; ^(*p*)^provisional. *2018; ^†^27 countries.

^1^ Malta–Germany.

^2^ Bulgaria–Luxembourg.

^3^ Italy–Ireland.

^4^ Bulgaria–Cyprus.

^5^ Bulgaria–Italy.

^6^ Italy–Ireland.

^7^ Estonia–Malta.

### Meningococcal epidemiology

In 2019, in the EU/EEA member states, the IMD NR was 0.57/100,000: 7.24/100,000 for infants, 2.00/100,000 for toddlers, and 0.88/100,000 cases for young people.

**Figure 1:**
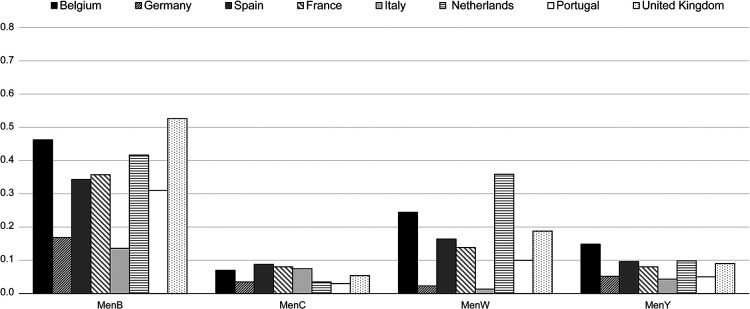
Notification rate of confirmed cases* (*N*/100,000) by country for each serogroup in 2019 (21). BE, Belgium; DE, Germany; ES, Spain; FR, France; IT, Italy; NL, Netherlands; PT, Portugal; UK, United Kingdom. *Confirmed case of IMD is defined as any person meeting at least one of the following laboratory criteria: isolation of *N. meningitidis* from a normally sterile site, or purpuric skin lesions; detection of *N. meningitidis* nucleic acid from a normally sterile site, or purpuric skin lesions; detection of *N. meningitidis* antigen in cerebrospinal fluid; detection of Gram-negative stained diplococcus in cerebrospinal fluid.

**Figure 2:**
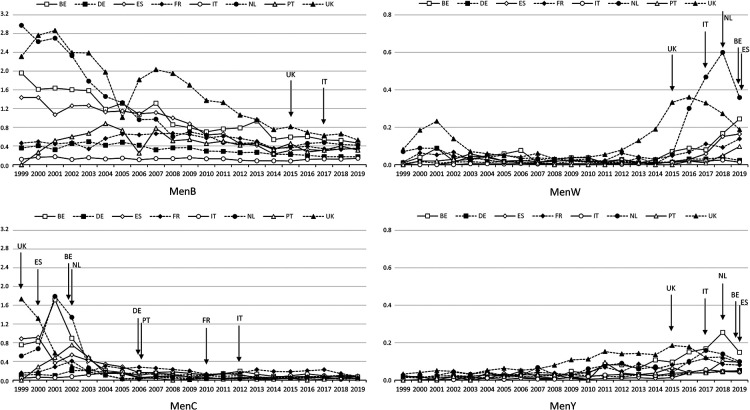
Notification rates of confirmed cases* (*N*/100,000) from 1999^†^ to 2019 by country and by serogroup. BE, Belgium; DE, Germany; ES, Spain; FR, France; IT, Italy; NL, Netherlands; PT, Portugal; UK, United Kingdom. *Confirmed case of IMD is defined as any person meeting at least one of the following laboratory criteria: isolation of *N. meningitidis* from a normally sterile site, or purpuric skin lesions; detection of *N. meningitidis* nucleic acid from a normally sterile site, or purpuric skin lesions; detection of *N. meningitidis* antigen in cerebrospinal fluid; detection of Gram-negative stained diplococcus in cerebrospinal fluid. ^†^No data for PT in 1999. Arrows indicate vaccination introduction; pattern fills indicate the country introducing vaccination in its vaccination program.

**Figure 3:**
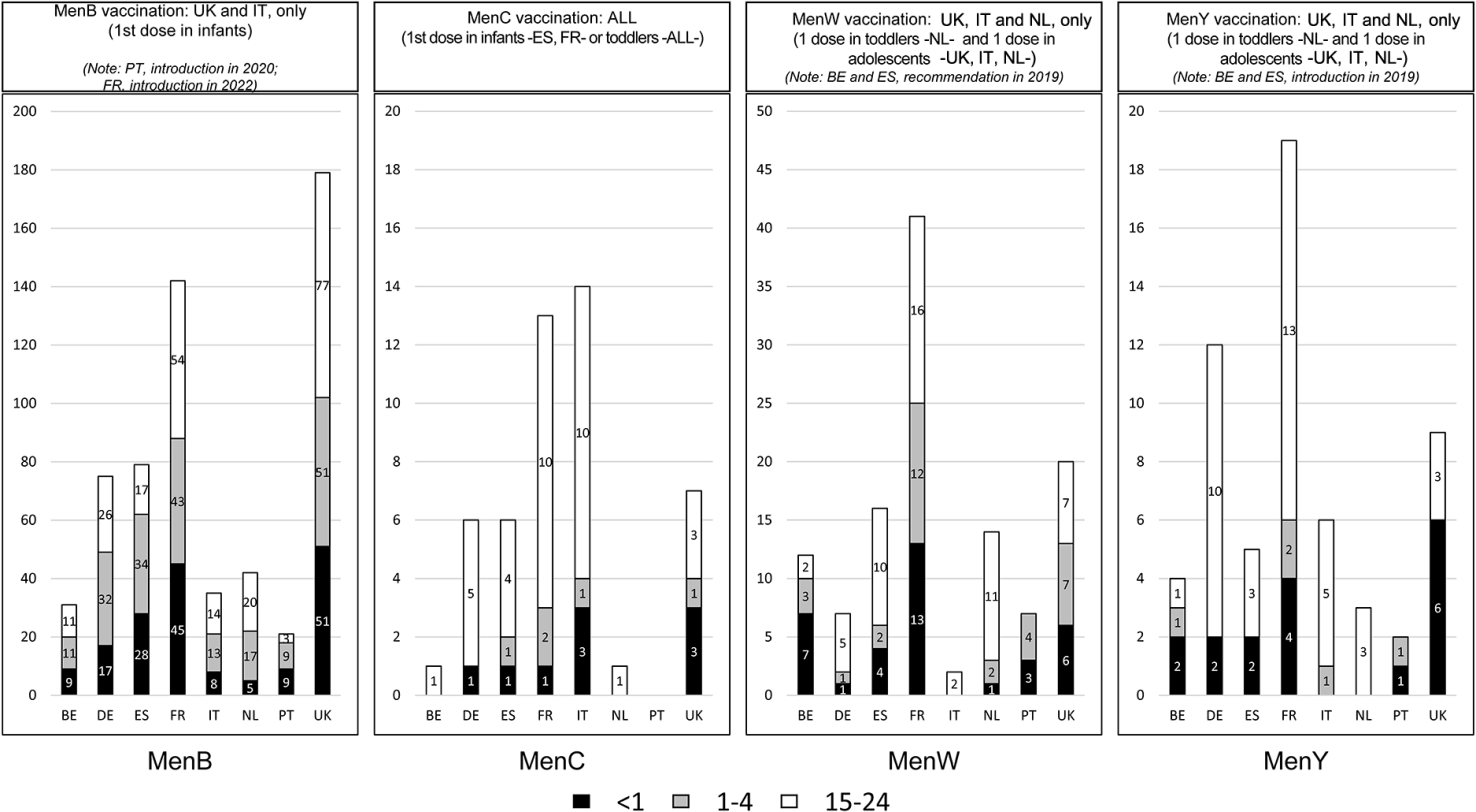
Number of IMD cases in infants, toddlers, and young people (15–24 years) in 2019 by country and serogroup. BE, Belgium; DE, Germany; ES, Spain; FR, France; IMD, invasive meningococcal disease; IT, Italy; NL, Netherlands; PT, Portugal; UK, United Kingdom. For each serogroup, information on vaccination with monovalent MenB vaccine, monovalent MenC vaccine, or quadrivalent MenACWY vaccine is provided for each country.

With an NR of 0.27/100,000, MenB was the most common serogroup overall and in all countries (Figure 1). Four (4) countries (BE, ES, NL, UK) had NR >1.0/100,000 in 1999, subsequently decreasing to levels comparable to other countries: i.e., ranging from 0.14/100,000 in Italy to 0.53/100,000 in the UK (Figure 2). NRs tended to be stable with respect to 2018 and 2017. The number of MenB cases in infants (<1 year) was particularly high in Spain (*N *= 28), France (*N *= 45), and the UK (*N *= 51) (Figure 3). Since 2003, MenC was at a low level in all countries (Figure 2). The NR was 0.06/100,000 in Europe as a whole. The highest NR was reported in Spain (0.09 per 100,000) (Figure 1). NR sharply decreased in 4 countries (BE, ES, NL, UK) over the 1999–2003 period in relation with the introduction in MenC conjugated vaccine NIPs. All other countries introduced routine MenC vaccination in their NIPs between 2006 and 2012 (Figure 2). In 2019, most cases occurred in adolescents and young adults aged between 15 and 24 years, in all countries except in the UK (Figure 3). No MenC cases were detected in 2019 in Portugal in the studied age groups. The total number of MenC cases was very low in all studied countries.

With an NR of 0.10/100,000, W was the second most frequently reported serogroup in Europe. The highest NR was reported in the Netherlands (0.36/100,000) and the lowest in Italy (0.01/100,000) (Figure 1). MenW NR remained low over time in the studied countries (Figure 2), although it rose clearly in the UK from 2013 and in the Netherlands from 2015; more recent smaller increases were reported for Belgium, Spain, France and Portugal (Figure 2). In 2019, in these 6 countries, serogroup W was the second most common serogroup (Figure 1). MenW cases occurred in all age groups (<1, 1–4, 15–24 years) except in Italy and Portugal (Figure 3).

MenY NR averaged 0.06/100,000 in 2019 in Europe, ranging between 0.04/100,000 and 0.15/100,000. The annual MenY NR remained low over time in the studied countries, with only slight variations over time (Figure 2); however, there was a slight increasing trend in all countries, notably in Belgium in 2018 (Figure 2). In 2019, most cases occurred in adolescents and young adults aged between 15 and 24 years, in all countries except Belgium, Portugal and the UK (Figure 3).

### Meningococcal vaccination

#### National immunization program (NIP)

Routine meningococcal prevention strategies were introduced between 1999 and 2012, starting with MenC vaccine. Routine strategies were then updated on 1 occasion in 3 countries (BE, IT, NL), 2 occasions in France and Portugal, 3 occasions in Spain, and 4 occasions in the UK. The German strategy has remained unchanged since its introduction in 2006 (Table 2).

**Table 2:** Introduction of meningococcal vaccines in the national vaccination programs and changes over time.

Country		First introduction of IMD vaccine*		Updates
BE	2002	1 dose at 15 months of age	2019	Switch from MenC to MenACWY for toddlers at 15 months of age Addition of booster dose of MenACWY vaccine for adolescents (15–16 years of age), with catch-up until 19 years of age
DE	2006	1 dose at 12 months of age Catch-up until 18 years of age	–	
ES	2000	3 doses: 2, 4 and 6 months of age Catch-up until 19 years of age	2005	Replacement of the MenC dose at 4 months of age by a dose at 12–18 months of age
			2013	Change from 2 MenC doses at 2 and 6 months of age to 1 dose at 4 months of age Addition of booster dose of MenC vaccine for adolescents (12 years of age), with catch-up until 18 years of age
			2019	Switch from MenC to MenACWY for the booster dose at 12 years of age
FR	2010	1 dose at 12 months of age	2017	Addition of 1 MenC dose at 5 months of age
		Catch-up until 24 years of age	2022	*Addition of MenB for infants and toddlers (3, 5, and 12 months of age)*
IT	2012	1 dose at 13–15 months of age Catch-up from 11 to 18 years of age	2017	Addition of booster dose of MenACWY vaccine for adolescents (12–18 years of age) *Addition of MenB for infants and toddlers (3, 4, 6, and 13 months of age)*
NL	2002	1 dose at 14 months of age Catch-up until 18 years of age	2019	Switch from MenC to MenACWY for toddlers at 14 months of age Addition of booster dose of MenACWY vaccine for adolescents (14 years of age), with catch-up until 18 years of age
PT	2006	1 dose at 3, 5, and 15 months of age	2012	Replacement of the MenC dose at 3,5, and 15 months of age by a dose at 12 months of age
			2020	*Addition of MenB for infants and toddlers (2, 4, and 12 months of age)*
UK	1999	3 doses at 2, 3 and 4 months of age, respectively	2006	Replacement of the dose at 2 months of age by a dose at 12–13 months of age, with catch-up until 25 years of age
		Catch-up until 18 years of age	2013	Removal of MenC dose at 4 months of age, with catch-up for toddlers until 5 years of age Addition of booster dose of MenC vaccine for adolescents (13–14 years of age), with catch-up until 25 years of age
			2015	Switch from MenC to MenACWY for the adolescent booster dose at 13–14 years of age *Addition of MenB for infants and toddlers (2, 4, and 12 months of age)*
			2016	Removal of the MenC dose at 3 months of age

BE, Belgium; DE, Germany; ES, Spain; FR, France; IMD, invasive meningococcal disease; IT, Italy; Men, meningococcal vaccine; NL, Netherlands; PT, Portugal; UK, United Kingdom; –, Not applicable.

* MenC was the first meningococcal vaccine introduced in the vaccination program of all countries.

In italics, addition of MenB in the national immunization program.

**Figure 4:**
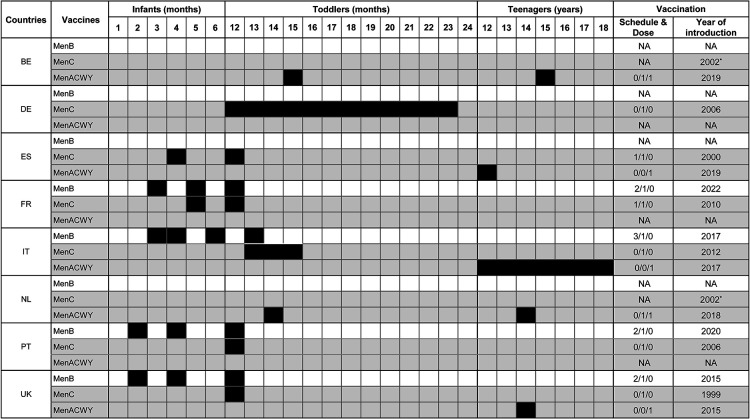
National recommendations for meningococcal vaccination in 2022 in selected European countries. BE, Belgium; DE, Germany; ES, Spain; FR, France; IT, Italy; NA, not applicable; NL, Netherlands; PT, Portugal; UK, United Kingdom. *Since then, switch from MenC to MenACWY. Vaccination schedules are presented by age group (infants, toddlers, or teenagers). Figures (n/n/n) indicate the number of doses of each vaccine per period. Catch-up programs are not mentioned. MenB: protein-based vaccine (white); MenC or MenACWY: conjugate vaccine (gray). Black boxes indicate age at vaccination. For example, in the UK, the MenC dose is administered at 12 months of age, and in Germany between 12 and 23 months.

Meningococcal C vaccines were introduced in the late 1990s early 2000s, first in the UK then in Belgium, Spain, and the Netherlands (Figure 4). At the time of the introduction, these countries were facing an increase in incidence, and the NIP updates led to a sharp decrease (Figure 2). Other countries (DE, FR, IT, PT) started vaccination programs between 2006 and 2012, unrelated to any specific increase in incidence at the time of introduction. All studied countries had a MenC NIP in place by 2012.

MenB recombinant protein vaccines were first introduced in the UK in 2015 and then in Italy in 2017 (Figure 4). At introduction, the UK showed the highest incidence and Italy the lowest (Figure 2). In both countries, incidence was stable or decreasing (Figure 2). MenB recombinant protein vaccine was then introduced in Portugal in 2020 and in France in 2022. At the time of writing, no other country had a routine MenB vaccination program.

As a result of increased MenW incidence worldwide, including Europe, several countries used quadrivalent MenACWY vaccines (Figure 4). The UK and then the Netherlands introduced a MenACWY vaccination program in 2015 and 2018, respectively, as an emergency measure to counter the rise in MenW incidence (Figure 2). The UK introduced MenACWY in adolescents along with a MenB program in infants and toddlers, whereas the Netherlands introduced MenACWY for toddlers and teens. Belgium and Spain, where MenW incidence was rising, recommended vaccination programs with MenACWY in 2019 (no implementation in Belgium at the time of writing), whereas Italy started vaccination with MenACWY in 2017 with no link to any increase in incidence. To date, France and Germany have not recommended routine MenACWY vaccination.

As of July 19, 2022, all countries covered MenC risk in one way or another: 3 *via* MenC monovalent vaccines for infants (FR) or toddlers (DE, FR, PT) with no adolescent program, 3 with a mix of MenC for infants (ES) and toddlers and MenACWY for adolescents (ES, IT, UK), and 2 *via* MenACWY vaccines for toddlers and adolescents (BE, NL) (Figure 4).

#### Vaccination coverage rate (VCR)

In 2021, in countries with only MenC programs, by the age of 24 months (2 years), 91% and 80% of children were vaccinated with MenC conjugate vaccine in France and Germany respectively. In Germany, 90% of 4–7-year-old children were vaccinated against MenC. In Portugal, in 2019, the VCR was 99% in 2-year-old children (30).

In 2019, in countries with MenACWY vaccine programs, 91% and 94% of 2-year-olds received MenC vaccine in the UK and in Spain, respectively. In the Netherlands (where 2019 was the year of the NIP update from MenC to MenACWY), 93% of toddlers were vaccinated against MenC, including 9% *via* a MenACWY vaccine. Finally, in Italy, 79% of children aged 24 months were vaccinated against MenC, including 47% *via* a MenACWY vaccine. Regarding adolescents, in the UK, 87% of adolescents received a MenACWY vaccine in 2019. In Spain and the Netherlands, NIPs were updated from C to ACWY vaccine that same year, with a catch-up program for the latter: 89% of adolescents in Spain were vaccinated against MenC (including 13% *via* a MenACWY vaccine); 86% of adolescents in the Netherlands received a MenACWY vaccine. Finally, in Italy, 75% of adolescents aged 16 but 58% of 18-year-olds received a MenACWY vaccine.

Regarding MenB, in 2019, 69% and 90% of children aged 24 months were vaccinated in Italy and the UK, respectively. No data were available for Portugal or France, as vaccination was not introduced until 2020 and 2022, respectively.

## Discussion

The WHO “Defeating meningitis by 2030” global roadmap aims to reduce cases of vaccine-preventable bacterial meningitis by 50% and deaths by 70% (19). Although it is now a vaccine-preventable disease, IMD remains a public health concern given the possibility of outbreaks and its unpredictability, morbidity, and mortality (≈10%). Despite appropriate effective antibiotic treatments and tremendous efforts to improve care, the rates of IMD mortality and of patients with severe neurological and functional sequelae have remained stable in recent years (12), partly because early clinical signs may be mild and/or nonspecific, making diagnosis difficult, with rapid disease progression.

In Europe, there is no uniform IMD immunization program except in epidemic situations (31). In some countries, IMD vaccination is part of the general NIP, whereas in other countries it is only recommended for specific groups (20). Some countries implement a vaccine strategy aiming at individual protection whereas others also target indirect protection and herd immunity provided by conjugate polysaccharide vaccines. The present study confirms the heterogeneity of NIPs, even in neighboring countries, whether with similar or different epidemiology, indicating that NIPs are also determined by other factors than epidemiology.

The increase in MenC incidence at the end of the 1990s was the major determinant of the introduction of MenC vaccine for infants and children in several EU countries (7). Introduction of MenC vaccination **in the UK** resulted from epidemiological considerations, starting in November 1999, facing high fatality rates due to the rapid expansion of a hypervirulent clone belonging to cc11. The NIP included a 3-dose vaccination schedule (2, 3, and 4 months of age) without booster dose, and a catch-up campaign (1 dose in children aged 1 to 18 years and then up to 25 years). The VCR reached ≈85% (88% for infants and 96% for children) within 18 months following the start of the vaccination campaign, and MenC incidence decreased by more than 80% in the targeted population (32, 33). **In the Netherlands**, MenC vaccination was introduced in the light of epidemiological factors and attempted to strike a balance in terms of dose number and timing of administration. In 2000–2001, confronted by similar epidemiology, the Netherlands adopted the UK model, although vaccination was implemented differently: vaccination (1 dose) of all 14-month-old toddlers and a catch-up campaign for all 1- to 18-year-old children, in order to target the meningococcal reservoir age group (adolescents). VCR rapidly reached 94%, and a 92% reduction in MenC incidence (99% in children aged between 1 and 18 years) was observed 2 years after introduction of MenC vaccination in the NIP. The decreased MenC IMD incidence in children aged <1 year (-92%) and >18 years (-80%) also supported a collective protective effect beyond the targeted population (herd immunity). This drastic NIP impact was attributed to the high VCR, particularly in adolescents, who are the reservoir for meningococcal carriage (34, 35). **In France**, MenC vaccination was recently introduced for epidemiological reasons, and was then drastically changed to cope with “real world” constraints. In 2002, in France, MenC incidence (0.6/100,000) was below the level expected to trigger introduction of IMD vaccination in the NIP. A few years later, thanks to the vaccination programs, MenC incidence had decreased in most European countries, leaving France among the top countries for MenC incidence (0.26/100,000) and leading to the introduction of MenC vaccination in 2010 (36). The French NIP attempted to follow the Dutch model: 1 dose at 12 months of age and a catch-up campaign in all children, adolescents and adults <24 years of age (targeting the reservoir to reach herd immunity, as in the Netherlands). In 2015, five years after the introduction of routine MenC vaccination, VCRs were low in adolescents and young adults: 23% for adolescents aged 15 to 18 years and 6.6% in young adults aged 20 to 24 years. Whereas rapid catch-up campaigns for adolescents in the Netherlands showed the impact of indirect protection, no herd immunity was obtained, no sterilization of the reservoir was reached, and ultimately, the NIP failed in France regarding MenC incidence (37). According to Taha et al. (20), the failure could be due to vaccine hesitancy, with lack of active efforts to apply and explain the vaccination strategy. In 2017, the French National Health Authority therefore recommended the addition of 1 dose of MenC vaccine at 5 months of age to complement the 12-month dose (38), which increased the initial cost of the program. Both vaccine doses became part of the mandatory NIP for infants and toddlers implemented as of 2018. As a result, the VCR quickly increased above the 85% threshold (27), leading to a decrease in the number of MenC cases in infants, the age group with the highest incidence in France (20).

Non-epidemiological factors may also underlie NIPs. Reviewing methodologies, frameworks and decision-making processes for economic evaluations of vaccines, with a focus on evaluation of vaccines targeting IMD, Christensen et al. (39) showed that evaluation of vaccination decision criteria varied between countries. According to the authors, all countries considered clinical outcome and cost-effectiveness, most countries considered disease burden and national health system priorities, some countries considered equity and budget impact, and a few considered peace-of-mind benefits and public or social preferences. **In the UK**, in July 2013, the Joint Committee on Vaccination and Immunization (JCVI) issued an interim position statement for consultation that did not advocate introducing MenB vaccine, based on cost-effectiveness evaluation (40), whereas the finalized statement introduced the MenB vaccine in the NIP. Despite its cost and unfavorable cost-effectiveness, MenB vaccination was proposed for all infants. The new vaccination program was implemented in 2015 at a time when MenB incidence was falling (41). The decision was welcomed by the meningitis patients' associations, supported by vaccine manufacturers and health care professionals who had advocated prevention. **In France**, vaccination of infants against MenB was initially not recommended (and not reimbursed under the national health insurance scheme) for all children by the National Health Authority. In February 2021, the National Health Authority recommended not changing the vaccination strategy, mainly because MenB cases were uncommon and based on the absence of epidemiological increasing trend and on an unfavorable cost-effectiveness analysis (42). However, after public consultation and strong advocacy from the representatives of scientific societies (primarily pediatric and infectious disease societies) the analysis was modified by the Health Authorities in June 2021, leading to the recommendation to vaccinate all children <24 months of age. The factors that changed the decision were: (i) long-term sequelae, which are largely underestimated; (ii) the potential risk of a rebound in pediatric infectious diseases when non-pharmaceutical interventions against the COVID-19 pandemic are no longer be applied; and (iii) social inequalities (43). In this regard, offering effective prevention against a severe pediatric disease only to families that can afford the vaccine would have been unethical, especially as low family income increases the risk and severity of the disease (4, 44). However, the National Health Authority re-emphasizes that the cost of this vaccination is high compared to its expected benefits, based on the results of the French cost-effectiveness study by Lecocq et al. (43, 45). In their study (45), the authors showed that routine vaccination against serogroup B meningococcal disease was not cost-effective given the current meningococcal epidemiology in France and protection data provided for the MenB vaccine. **In Italy**, MenC (2012) and MenB (2017) vaccinations have been included in the NIP, although incidence of both serogroups is extremely low, suggesting that the Italian NIP program is more “preventive” than “curative” from a public health perspective.

IMD is an uncommon but serious disease, with possible long-term sequelae. According to a recent French study in real-life conditions, a quarter of cases of IMD presented at least 1 sequela and these patients generated a disproportionate amount of the cost, both for initial hospitalization and for costs accrued over the following years (4). Belonging to a family with low income was identified as a risk factor for serious IMD (46). Regardless of the combination of factors driving the NIP updates, an appropriate vaccination strategy with the right vaccines administered in the right target population with a high VCR has significant impact, as demonstrated with MenC (47). Inclusion of IMD vaccination in the NIP is therefore an appropriate way to promote equitable prevention in all social categories. Therefore, in the light of this study and the diversity of meningococcal vaccination schedules in France and neighboring countries, a convergence towards a common optimal IMD immunization program in Europe (as done for COVID-19) would be advisable and likely to improve understanding by patients and healthcare professionals. Within the passport-free Schengen zone, a common strategy would allow children and adolescents to travel between neighboring countries more safely. Demographic interaction is frequent in Europe (20). In 2019, there were 93.1 million visits overseas by UK residents (Spain, France, Italy, the Netherlands and Germany being in the top 10 destinations) and conversely there were 40.9 million visits to the UK (France, Germany, Spain, Italy, the Netherlands and Belgium, being in the top 10) (48). However, such a common vaccination schedule would run up against real-life problems, including the diversity of primary healthcare for children in Europe. A survey published in 2010 (49) showed that the timing and number of scheduled healthcare consultations for children varied greatly between countries. For example, the mean number of clinical consultations for well-child check-ups was 14.7 but ranging from 5 to 30 according to the country (*N *= 29). In addition, in most countries, the vaccination schedule follows the school cycle; differences in school systems could explain the differences observed in the age groups targeted by the NIPs. Another limitation is the differences in governance between EU countries. NIPs are usually organized at national level, whereas the regional level tends to be in charge of overseeing implementation of vaccination and monitoring VCR (50). However, **in Belgium**, the vaccination program is organized at subnational level, and **in Germany** and **Spain** regional levels are able to adapt the national vaccination program. As a result, vaccination programs may differ between Belgian communities and German and Spanish regions. For example, in Spain, vaccination in infants/toddlers and adolescents used MenC vaccine until 2019. In 2019, vaccination with MenACWY vaccine was introduced for adolescents, leading to switch from MenC to MenACWY in this population. However, 2 regions (Castilla y Leon and Andalucía) also decided to switch from MenC to MenACWY for toddlers (12–15 months). In Germany, the NIP has included MenC in toddlers since 2006 but, in the region of Saxony, the Saxon Vaccination Commission (SIKO: *Sächsische Impfkommission*) decided to switch to MenACWY for infants/toddlers, and has additionally recommended a booster for adolescents since January 2019. A similar stepwise approach is seen in **Italy**: since the authorization of MenB vaccine by the European Medicines Agency (EMA) in 2013, and prior to the introduction of the vaccination in the Italian NIP in 2017, MenB vaccine was provided free of charge in a few Italian regions (51). It can be hypothesized that regional immunization programs impact the NIP, increasing reactivity and spreading changes in NIP.

The present study had some limitations. Due to the data set, it did not analyze barriers delaying or precluding changes in NIP or slowing down new NIP implementation, such as the time taken by the National Immunization Technical Advisory Group (NITAG) to issue vaccination recommendations, or the time needed to move on from recommendations to effective access for the population (52). In addition, country-specific factors could not be analyzed. The complete vaccination path (from initial prescription to administration, through vaccine dispensing) is complex to analyze, as steps and places of administration vary between countries. For example, in France, 2 medical consultations are required for vaccination (prescription, then administration), which may impact the VCR, especially for adolescents. Vaccine prices and partial national insurance cover, leaving out-of-pocket expenses, vary between countries and impact VCR. Data have been analyzed from 8 selected EU countries. However, (i) the selected countries have variable vaccination strategies that could impact the availability of newly developed vaccines and their introduction in the NIPs; (ii) they represent more than two-thirds of the EU-28 population; (iii) interactions between countries are frequent; (iv) their demographic characteristics (e.g., fertility rate, proportion of young people) differ greatly (22), which could explain differences in how children are perceived in society. In addition, the selected countries all had similar mean socioeconomic levels, to avoid bias linked to economic factors influencing recommendations, although NITAG processes should focus on providing the most appropriate prevention program, regardless of assessment of pricing and national insurance cover (52). Vaccine prices are variable in Europe and depend on the way contracts with pharmaceutical companies are awarded (tenders or private markets). This heterogeneity complicates the analysis of decision-making process; moving toward a single European price (as was done for COVID-19 vaccines) could help. Epidemiological data were collected before the COVID-19 pandemic. Since the implementation of non-pharmaceutical interventions as pandemic control measures, IMD incidence and associated mortality fell across various regions, including European countries (53). However, the COVID-19 situation should not slow vaccination program updates, especially since meningococcal transmission could be facilitated by the lifting of the measures that minimized close contact and limited social gatherings and the reduction in childhood vaccination rates observed in some countries during the pandemic (54). According to a preprint analysis by the UK Health Security Agency, there was an increase in MenB IMD in adolescents and young adults in England following the easing of COVID-19 containment measures (55).

In conclusion, the present study confirmed the diversity of NIPs, even in neighboring countries with similar factors like economic resources and epidemiological risk, highlighting other factors driving NIPs. Although various factors are considered for updates, current NIPs should aim at prevention against circulating serogroups using MenB and MenACWY vaccines. Convergence toward a common European immunization program would improve equity between countries, promote safe travel regarding infectious diseases in the European area, and possibly increase understanding among healthcare professionals and the lay public. For example, European convergence on an IMD immunization program could start with MenACWY vaccination in adolescents, continue with general vaccination of infants and toddlers against serogroup B, and extend to vaccination against other serogroups (ACWY) in this age group. A consensual approach would be required to overcome expected implementation difficulties, as was successfully done for COVID-19 vaccination.

## Data Availability

The original contributions presented in the study are included in the article/**Supplementary Material**, further inquiries can be directed to the corresponding author/s.

## Author’s note

All named authors attest that they meet the International Committee of Medical Journal Editors (ICMJE) criteria for authorship for this article, take responsibility for the integrity of the work as a whole, and have given their approval for this version to be published. Medical writing support was provided by Alexiane Baloche, and Abelia Science (Saint-Georges-sur-Baulche, France).
