# Supplementary material for: Overview of meningococcal epidemiology and national immunization programs in children and adolescents in 8 Western European countries
Source: Front Pediatr. 2022 Nov 23;10:1000657. doi: 10.3389/fped.2022.1000657 (PMC9727280; doi:10.3389/fped.2022.1000657)
Supplement: Supplementary file 1 [file Table1.docx]

| **Epidemiology** | | | |
| --- | --- | --- | --- |
| All | ECDC | (21) | Confirmed case, notification rate  (/100,000 inhabitants) |
| **Vaccine schedule** | | | |
| All (except UK) | ECDC | (21) |  |
|  |  |  |  |
| UK | Gov.uk | (23) |  |
| **Vaccination coverage** | | | |
| Belgium  (BE) | Sciensano | (24) | The immunization coverage is based on public surveys data collected in each region (Flanders, Wallonia, Brussels). A person is considered vaccinated when the desired immunization status has been achieved on schedule for a certain individual age according to the national immunization schedule. |
| Germany (DE) | Robert Kock Institut | (25) | The immunization coverage is based on the school entrance data and the billing register for vaccine acts. A person is considered vaccinated when the desired immunization status has been achieved on schedule for a certain individual age according to national immunization schedule. |
| Spain (ES) | Ministerio de Sanidad | (26) | The immunization coverage is based on the administrative data base. Percentage of individuals vaccinated with one dose of MenC vaccine after 12 months of age for children, or with one dose of MenACWY vaccine after 10 years of age for adolescents. |
| France (FR) | Santé publique France | (27) | The immunization coverage is based on the French national insurance information system (SNIIRAM). It is the percentage of children vaccinated with one dose of MenC vaccine. |
| Italy (IT) | Ministero della Salute | (28) | The immunization coverage is based on data collected from autonomous province and region and analysed by the general management of sanitary prevention. A person is considered vaccinated when the desired immunization status has been achieved on schedule for a certain individual age according to the national immunization schedule. |
| The Netherlands (NL) | National Institute for Public Health and the Environment | (29) | The immunization coverage is based on the national registration system and the database of municipal personal files. A person is considered vaccinated when the desired immunization status has been achieved on schedule for a certain individual age according to national immunization schedule. |
|  |  |  |  |
| Portugal | Instituto Nacional de Estatística | (30) | Vaccination coverage to individuals who complete 2 years of age (2017 - %) by Place of residence (NUTS II - 1999) and Type of administrated vaccine; Annual - Directorate-General of Health |
|  |  |  |  |
| The United Kingdom (UK) | Gov.uk | (23) | Data were received from all Health Boards (HBs) in Scotland, Northern Ireland, and Wales. In England, Local Teams (LTs) and Child Health Record Departments (CHRDs) provided data for all upper tier local authorities (LAs). The cover of vaccination evaluated rapidly programme (COVER) evaluates childhood immunization in England, collating data for children aged 1, 2 and 5. |
| **Vaccine** |  |  |  |
| All | EMA | (18) | |

ECDC: European Centre for Disease Prevention and Control; EMA: European Medicine Agency; NUTS: Nomenclature of territorial units for statistics; UK: United Kingdom.

* Confirmed case of IMD is defined as any person meeting at least one of the following laboratory criteria: isolation of *N. meningitidis* from a normally sterile site or purpuric skin lesions; detection of *N. meningitidis* nucleic acid from a normally sterile site or purpuric skin lesions; detection of *N. meningitidis* antigen in cerebrospinal fluid; detection of Gram-negative stained diplococcus in cerebrospinal fluid (1).
